# Effect of under-nutrition during pregnancy on low birth weight in Tigray regional state, Ethiopia; a prospective cohort study

**DOI:** 10.1186/s40795-021-00475-7

**Published:** 2021-11-16

**Authors:** Kidanemaryam Berhe, Lemlem Weldegerima, Freweini Gebrearegay, Amaha Kahsay, Afewerki Tesfahunegn, Mehammedseid Rejeu, Brhane Gebremariam

**Affiliations:** 1 Department of Nutrition and Dietetics, School of Public Health, College of Health Sciences, Mekelle University, Mekelle, Tigray Ethiopia; 2 Department of Epidemiology, School of Public Health, College of Health Sciences, Mekelle University, Mekelle, Tigray Ethiopia; 3 Department of Maternity and Reproductive Health Nursing, School of Nursing, College of Health Sciences, Mekelle University, Mekelle, Tigray Ethiopia; 4 Tigray Institute of Policy Studies, Mekelle, Tigray Ethiopia

**Keywords:** Under-nutrition, Pregnant women, Incidence, Low birth weight, Tigray

## Abstract

**Background:**

Under-nutrition during pregnancy affects birth outcomes and neonatal outcomes. Worldwide, 20.5 million children were low birth weight, mainly in poor countries. However, there is no longitudinal-based evidence on the effect of under-nutrition during pregnancy on birth weight in Tigray regional state. Therefore, this study aimed at investigating the effect of under-nutrition during pregnancy on low birth weight in Tigray regional state.

**Methods:**

We conducted a prospective cohort study among consecutively selected 540 pregnant women attending antenatal care in hospitals from October 2019 to June 2020. Pregnant women with mid upper arm circumference (MUAC) < 23 cm were exposed and those with MUAC≥23 cm were unexposed. Data on socio-demographic, diet, hygiene and anthropometry measurements were collected using pretested and structured questionnaires. SPSS version 25 was used for analysis. A log-binomial model was used to estimate the adjusted risk ratio and its 95%CI of the risk factors for low birth weight. Multi-collinearity was checked using the variance inflation factor (VIF) at a cut-off point of 8 and there was no multi-collinearity.

**Result:**

The overall incidence of low birth weight was 14% (95%CI: 11.1, 17.4%). The incidence of low birth weight was 18.4 and 9.8% among the exposed and unexposed women, respectively. The difference in low birth weight incidence between the exposed and unexposed groups was statistically significant (*p*-value = 0.006). The risk factors of low birth weight were maternal illiteracy (ARR: 1.8, 95%CI: 1.01, 3.3), low monthly family income < 50 US Dollar (ARR: 1.6, 95%CI: 1.07, 2.2), lack of latrine utilization (ARR: 0.47, 95%CI: 0.28, 0.78), and diet diversity score < 5 (ARR: 1.9, 95%CI: 1.05, 2.61).

**Conclusion:**

Low birth weight was significantly higher among the exposed pregnant women. Maternal illiteracy, low monthly income, lack of latrine utilization, and low DDS were risk factors of low birth weight. It is then important to strengthen nutritional assessment and interventions during pregnancy, with a special attention for illiterate, and low monthly income pregnant women. Again, there has to be a promotion of latrine utilization and consumption of diversified diets.

## Background

In order to produce a healthy baby, maternal intake must supply all the nutrients needed to provide for the growth and development of the fetus. Under-nutrition during pregnancy affects birth outcomes, neonatal outcomes and later health outcomes of the offspring [1–3]. Maternal under-nutrition is a public health problem in Ethiopia [4]. According to the Ethiopian demographic and health survey (EDHS) 2016, 22% of reproductive age women in Ethiopia and 34% in Tigray regional state were undernourished (body mass index< 18.5 kg/m^2^). When undernourished women get pregnant, the fetus has an increased risk of low birth weight [5]. The causes of maternal under nutrition are complex and interrelated which includes inadequate dietary intake, diseases, food insecurity, inadequate care, unhealthy household environmental, lack of health services, poverty, lack of capital, social and political problems [6].

According to the World Health Organization (WHO), low birth weight is defined as weight at birth less than 2.5 kg(kg) [7]. Worldwide, 20.5 million live births were low birth weight (LBW), 91% were from low and middle-income countries, mainly southern Asia (48%) and sub-Saharan Africa (24%). Birth weight is affected by maternal diet, socio economic status, maternal health and nutrition, pregnancy complications, infection, and poverty [6, 8, 9]. The consequences of low birth weight are: increased fetal and neonatal mortality, morbidity, poor growth, impaired cognitive development, chronic disease later in life, and intergenerational under nutrition cycle [6, 7].

Despite of the establishment of food and nutrition policy in Ethiopia in 2018 [10] and the implementation of the national nutrition program since 2008 [11], the burden of under-nutrition and its consequences remain high. Recent findings showed that both neonatal and infant mortality rates are unacceptably high in Ethiopia including Tigray regional state [12]. In Tigray regional state (the study area), neonatal mortality and infant mortality rates were 34 and 43 per 1000 live births, respectively [5]. Investigating the effect of under-nutrition during pregnancy on low birth weight is important for evidence-based interventions to reduce the burden of low birth weight and its contribution to neonatal and infant mortality in Tigray. To our knowledge, there is no longitudinal based evidence on the effect of under-nutrition during pregnancy on low birth weight in Tigray regional state. Therefore, this study was aimed to investigate the effect of under-nutrition during pregnancy on low birth weight in Tigray regional state by applying a prospective cohort study design.

## Methods

### Study area, period and design

The study was conducted in Tigray regional state, Ethiopia. It is located at 780 km from the capital of Ethiopia, Addis Ababa. According to the 2018 Tigray regional health bureau report, there are 7 zones, 52 woredas/districts, 780 health posts, 227 health centers, and 18 hospitals. The major agricultural products found in Tigray regional state are cereals (Taff, barley, maize, and wheat), grains (bean, soya bean, and pea), vegetables, fruits, honey, and roots, animal products like meat, poultry, and milk, and milk products. We applied a prospective cohort study design from October 2019 to June 2020.

### Population, recruitment and follow up

The source population was all pregnant women attending antenatal care (ANC) in hospitals of Tigray regional state whereas the sample population was all pregnant women attending ANC in the randomly selected hospitals of Tigray regional state. The inclusion criteria were first ANC visit with gestational age not more than 16 weeks, willingness to attend routine ANC visits and permanent residence, and more than six months residency in Tigray regional state. Pregnant women with severe illness, overweight or obesity (BMI ≥ 25 kg/m^2^), and multiple pregnancies were excluded from the study. Pregnant women were recruited at first ANC contact (≤16 gestational weeks). Then, they were followed at ANC2 (20–26 gestational weeks), and ANC3 (30–40 gestational weeks). Finally, data on birth outcomes were taken during the institutional delivery. We had planned to exclude still births but such cases were not found during our data collection period.

### Sample size determination

In this prospective cohort study, the ratio of exposed to unexposed pregnant women group was 1:1. We calculated the sample size using Epi-Info version 7.2.4 with the assumptions of 95% significance level (2-sided), 80% power. Besides, we took the incidence of low birth weight for exposed (11.8%) and unexposed (10.4%) pregnant women, and a relative risk of 1.9 from a study conducted in Tigray regional state in 2014 [13]. Then, we considered a 10% loss to follow up and our total sample size for this study was 540 (270 for exposed and 270 for the unexposed pregnant women).

### Sampling technique

Firstly, we used a simple random sampling technique to select a total of six hospitals (Mekelle hospital Wukro hospital, Adigrat hospital, Adwa hospital, Aksum hospital, Suhul hospital) from a total of 18 hospitals in Tigray regional state. Secondly, we took a total number of study participants from each hospital based on their proportion to population size (PPS) i.e. proportional to their average client size attended per month by referring to the registration books of each antenatal care unit. Lastly, we recruited participants at the antenatal care unit using a consecutive sampling technique until the required sample size was attained. The recruited pregnant women were followed until they gave births.

### Study variables

The dependent variable was the incidence of low birth weight and the independent variables include; socio-demographic and economic factors (age, educational status, marital status, parity, monthly income, residence, and religion), diseases (anemia, syphilis, hepatitis B virus, and HIV), dietary-related factors (diet diversity scores, food insecurity, drinking of tea/coffee), hygiene and sanitation-related factors (latrine, hand washing, and access to safe water), and anthropometry measurements (maternal height and weight, mid-upper-arm circumference (MUAC), gestational weight gain, and newborn weight). Gestational age at first contact was determined by the last normal menstruation period (LNMP), fundal height measurement, and or ultrasound. Low birth weight is weight < 2.5 kg at birth [7]. MUAC was used to assess the nutritional status of pregnant women. Those with a MUAC value less than 23 cm were considered as undernourished (exposed) and those with MUAC≥23 cm were normal nourished (unexposed) [14, 15]. Diet diversity scores (DDS) of the pregnant women were calculated using the 24-h dietary recall method. DDS is a proxy indicator for the quality of consumed diet, which in turn reflects the consumption of micronutrients. DDS out of 10 points was computed by combining the values of all the groups. The DDS was categorized as low (< 5) and recommended (≥5) [16]. Household food insecurity scores were classified into one of the four categories: food secured, mildly food insecure, moderate food insecure, and severely food insecure [17].

### Data collection tools, procedures and quality control

Data were collected by face-to-face interview using a pre-tested and structured questionnaire developed from EDHS and other literature [5, 12, 13, 16, 18]. Initially, the questionnaire was prepared in English and contextualized/adapted in a culturally relevant and comprehensive form. Then, it was translated into Tigrigna (local language) and translated back to English by language experts to check consistency. The questions were simple, clear and unambiguous. Nutrition and health experts have participated in developing and commenting the questionnaire. Some wording and sequences of the questions were modified after pre-test the questionnaire. The questionnaire had baseline questions concerning socio-demographic and economic, diseases, diet, hygiene, and sanitation-related factors and anthropometry measurements. The questionnaire had follow-up questions on MUAC, gestational weight gain, diet diversity score (DDS), and iron folate supplementation. Finally, the questionnaire had questions about birth outcomes like the sex of the newborn, live/dead, and birth weight.

Anemia was identified using the WHO hemoglobin concentration cutoff point, less than 11 g/dl [19]. There were counseling and testing services to diagnose HIV, syphilis and hepatitis B virus in all hospitals. MUAC was measured by non-stretchable measuring tape. A tape was fixed at the mid-point between the elbow and the shoulder (acromion and olecranon). The tape measure was placed around the non-dominant arm; usually the left arm. The weight of the mother was measured in kilograms with a weighing scale (Seca designed by Germany) and rounded off to the nearest 0.1 kg. The height of the mother was measured with a stadiometer (Seca-2000, mechanical height meter), without shoes, and rounded off to the nearest 0.1 cm. The weight of the baby at birth was measured in kilograms on digital baby scales (Seca 354 Hamburg, Germany). The infants were weighed wearing no clothing. To estimate DDS, the pregnant women were asked to recall all the food items consumed in the previous 24 h preceding the data collection date. Then, the reported food items were classified based on the ten food groups (Cereals, Pulses, Nuts and Seeds, Dairy, Meat, Eggs, Dark green leafy vegetables, other vitamin A-rich fruits and vegetables, other vegetables, and other fruits). Consuming a food item with a minimum of 15 g (one teaspoon) from any of the groups was assigned a score of “1” and a score of “0” was assigned if no or less than 15 g food was consumed. Household food insecurity was assessed by a tool adopted from food and nutrition technical assistance (FANTA). The tool contains 18 questions; the first 9 questions were answered by yes or no based on the occurrence of the condition in the past four weeks. If the answer was yes for the occurrence question, a question for frequency of occurrence was asked to determine whether the condition occurred rarely (once/twice), sometimes (three to ten times), or often (more than ten times) [17]. Standard procedures were used in anthropometry measurements. All anthropometric measurements were taken three times and the average was calculated to ensure reliability. Data collectors and supervisors were BSc midwives. Two days’ training was given for data collectors and supervisors. All instruments were calibrated regularly using standard measurements. Data collectors were under close supervision and the collected data were reviewed and checked for completeness, clarity and accuracy on a daily basis prior to data entry.

### Data analysis

We used a statistical package for social sciences (SPSS) version 25 to analyze the collected data. We cleaned the data by sorting and tabulating simple frequency tables. Low birth weight was dichotomized into 1 = Yes and 0 = No. Then, we computed descriptive statistics for the study variables. Categorical variables were reported using frequencies and percentages. We checked the normality for the distribution of continuous variables using the Shapiro-Wilk test. We applied crosstabs to estimate the cumulative incidence of low birth weight. Chi-square test was used to assess the significant differences in the cumulative incidence of low birth weight. The difference was considered statistically significant at *P*-value < 0.05. We used a log-binomial model to estimate the adjusted risk ratio and its 95% confidence interval (CI) of the risk factors for low birth weight.

## Result

### Socio-demographic and economic characteristics of the pregnant women

**Fig. 1:**
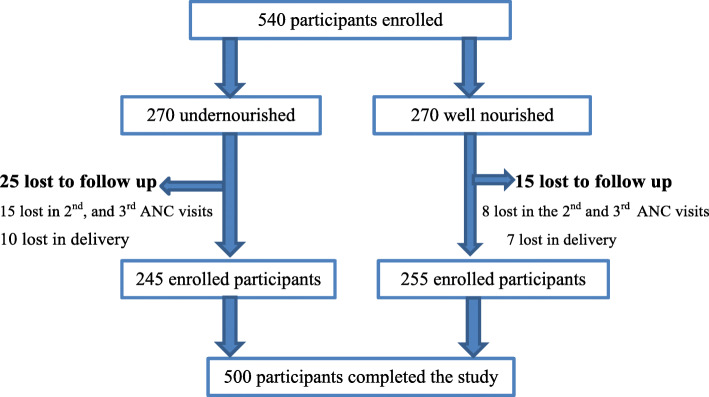
Study participants’ flow during the prospective cohort study of pregnant women in Tigray regional state, Ethiopia, 2020. [ANC: antenatal care]

Out of 540 participants, 500 (245 exposed and 255 unexposed) had completed and included in this study, with a total loss to follow up of 40 (7%) (Fig. 1).

The number of study participants of each randomly selected hospital at each visit is described in detail in the following table (Table 1).

**Table 1:** Number of participants during baseline (ANC1) and follow up visits (ANC2, ANC3, and delivery), Tigray regional state, Ethiopia, 2020

Name of hospitals		Number of study participants at each visit							
		ANC1 (≤16 Gestational weeks)		ANC2 (20–26 Gestational weeks)		ANC3 (30–40 Gestational weeks)		Delivery in hospitals	
		Planned	Actual	Planned	Actual	Planned	Actual	Planned	Actual
Mekelle hospital	Exposed	45	45	45	45	45	42	42	42
	Unexposed	45	45	45	45	45	44	44	43
	Total	90	90	90	90	90	86	86	86
Wukro hospital	Exposed	45	45	45	44	44	43	43	42
	Unexposed	45	45	45	45	45	44	44	42
	Total	90	90	90	89	89	87	87	87
Adigrat hospital	Exposed	45	45	45	45	45	43	43	40
	Unexposed	45	45	45	45	45	44	44	44
	Total	90	90	90	90	90	87	87	87
Adwa hospital	Exposed	45	45	45	42	42	42	42	40
	Unexposed	45	45	45	45	45	43	43	42
	Total	90	90	90	87	87	85	85	85
Aksum hospital	Exposed	45	45	45	45	45	43	43	42
	Unexposed	45	45	45	43	43	43	43	42
	Total	90	90	90	88	88	86	86	86
Suhul hospital	Exposed	45	45	45	43	43	42	42	39
	Unexposed	45	45	45	44	44	44	44	42
	Total	90	90	90	87	87	86	86	86
**Total**	Exposed	270	270	270	264	263	255	255	245
	Unexposed	270	270	270	267	266	262	262	255
	Total	540	540	540	531	531	517	517	500

*ANC1: Ante Natal Care 1, ANC2: Ante Natal Care 2, ANC3: Ante Natal Care 3*

The mean (±standard deviation) age of the participants was 28.05 ± 5.13 years. All the pregnant women in both groups were Tigrean in ethnicity. Majority of the pregnant women in the exposed group (84%) and almost all pregnant women in the unexposed group (96%) were Orthodox Christian in religion. Three-fourth (74.7%) of pregnant women among the exposed group and 218(85.5%) pregnant women among the unexposed group lived in urban areas. Majority of the pregnant women in both groups (95.1% of exposed and 91.4% of unexposed) were married. Educational status of the pregnant women and monthly income were significantly different between the groups (Table 2).

**Table 2:** Socio-demographic and economic characteristics of pregnant women in Tigray regional state, Ethiopia, 2020

Variables		Exposed group ***n*** = 245(%)	Unexposed group ***n*** = 255(%)	Chi-square test, ***P***-value
Age of the pregnant women	19–24 years	71 (29%)	53 (20.8%)	0.13
	25–29 years	56 (22.9%)	68 (26.7%)	
	30–35 years	80 (32.7%)	109 (42.7%)	
	Above 35 years	38 (15.5%)	25 (9.8%)	
Educational status of pregnant women	Illiterate	84 (34.3%)	43 (16.9%)	0.001
	Elementary (1–8 grades)	60 (24.5%)	65 (25.5%)	
	High school (9–10 grades)	57 (23.3%)	71 (27.8%)	
	Above high school	44 (18%)	76 (29.8%)	
Educational status of husbands	Illiterate	46 (19.7%)	26 (11.2%)	0.11
	Elementary (1–8 grades)	86 (36.9%)	57 (24.5%)	
	High school (9–10 grades)	51 (21.9%	66 (28.3%)	
	Above high school	50 (21.5%)	84 (36.1%)	
Occupation of pregnant women	Civil servant	15 (6.1%)	94 (38.5%)	0.09
	Daily worker/waitress/farmer	39 (15.9%)	11 (4.5%)	
	Own business	65 (26.5%)	13 (5.3%)	
	Housewife	126 (51.4%)	126 (51.6%)	
Occupation of husbands	Civil servant	72 (30.9%)	119 (51.1%)	0.21
	Own business	135 (57.9%)	92 (39.5%)	
	Daily worker/waitress/farmer	26 (11.2%)	22 (9.4%)	
Monthly income	< 50 US Dollar	78 (31.8%)	44 (17.3%)	0.001
	50–167 US Dollar	75 (30.6%)	106 (41.6%)	
	> 167 US Dollar	92 (37.6%)	105 (41.2%)	

### Hygiene and sanitation-related factors

Almost all pregnant women in both groups (94.7% in exposed and 98.1% in unexposed) were washing their hands at all critical conditions (after toilet, before food preparation, before eating, after cleaning child). Tap water was the water source for drinking for 87.8% of exposed pregnant women and 91.2% of unexposed pregnant women. One-third (68.4%) of the exposed group and three-fourth (74.9%) of the unexposed group were utilizing latrines. But, one-fourth (27.4%) pregnant women from the exposed group and 31(12.2%) of the unexposed group had no latrine. There was no significance difference of the variables between the groups.

### Dietary related factors

Twenty-eight (11.4%) pregnant women from the exposed group and 5(1.9%) pregnant women from the unexposed group reported foods forbidden to them (taboo) like meat, vegetable, and coffee. The reasons why the foods are forbidden are fear of infection, to prevent large babies, not to change the color (black mark on the body part) of the baby. Half (50.6%) of the pregnant women from exposed group and more than one-third (38.4%) of pregnant women from the unexposed group were in fast during the fasting period. Only two third pregnant women in both groups took iron-folate supplementation as told by the health professionals. Diet diversity score was low (< 5) in 160(65.3%) and 101(39.6%) exposed and unexposed pregnant women, respectively. One-fourth (27.8%) from exposed and 22(8.6%) from unexposed pregnant women had a history of alcohol drinking. Number of meals per day, number of additional meals per day and drinking coffee and/or tea with meals were significantly different between the groups (Table 3).

**Table 3:** Dietary related factors of pregnant women in Tigray regional state, Ethiopia, 2020

Variables		Exposed group (***n*** = 245)	Unexposed group (***n*** = 255)	Chi-square test, ***P***-value
Number of meals per day	≤3 times	203 (82.9%)	171 (67.1%)	0.001
	≥4 times	42 (17.1%)	84 (32.9%)	
Number of additional meals per day during pregnancy	No additional meals (as usual)	35 (14.3%)	78 (30.6%)	0.001
	Once	81 (33.1%)	46 (18%)	
	Twice	94 (38.4%)	72 (28.2%)	
	Three times and above	35 (14.3%)	59 (23.1%)	
Drinking of coffee/tea with meals or within 2 h of meal	No drinking of coffee or tea	58 (23.7%)	94 (36.9%)	0.005
	1–2 cups	99 (40.4%)	80 (31.4%)	
	≥3 cups	88 (35.9%)	81 (31.8%)	
Food security level	Moderate food insecure with hunger	17 (6.9%)	10 (3.9%)	0.14
	Mild food insecure without hunger	80 (32.7%)	72 (28.2%)	
	Food secured	148 (60.4%)	173 (67.8%)	

### Gynecological and obstetric related factors

Three fourth of the pregnant women in both groups (79.6% in the exposed group and 76.9% in the unexposed group) had no history of abortion in their previous pregnancies. More than half (55.5%) of the pregnant women in the exposed group and three-fourth (76.1%)) of the pregnant women in the unexposed group had vaginal bleeding in the index pregnancy. Thirty-nine (15.9%) pregnant women from the exposed group and 21(8.2%) pregnant women from the unexposed group had urinary tract infections. Variables between the groups were assessed for any significant difference but no variable was found statistically significant (Table 4).

**Table 4:** Gynecological and obstetric related factors of pregnant women in Tigray regional state, Ethiopia, 2020

Variables		Exposed group (***n*** = 245)	Unexposed group (***n*** = 255)	Chi-square test, ***P***-value
Type of modern family planning used before got pregnant	Not used	84 (34.3%)	42 (16.5%)	0.12
	Pills	0 (0%)	58 (22.7%)	
	Injectable	146 (59.6%)	118 (46.3%)	
	IUCD	0 (0%)	11 (4.3%)	
	Implant	15 (6.1%)	26 (10.2%)	
Total pregnancies including the index pregnancy	1	86 (35.1%)	46 (18%)	0.08
	2	58 (23.7%)	70 (27.5%)	
	3–5	73 (29.8%)	117 (45.9%)	
	≥6	28 (11.4%)	22 (8.6%)	
Type of pregnancy	Unplanned and unwanted	40 (16.3%)	36 (14.1%)	0.75
	Unplanned but wanted	55 (22.4%)	62 (24.3%)	
	Planned and wand wanted	150 (61.2%)	157 (61.6%)	
Previous uterus problems	No problem	210 (85.7%)	236 (92.5%)	0.41
	Discharge	15 (6.1%)	10 (3.9%)	
	Pelvic inflammatory pain	20 (8.2%)	9 (3.5%)	

*IUCD: intra-uterine contraceptive device.*

### Anthropometry measurements, laboratory findings, and delivery outcomes

Almost two-thirds (73.5%) of pregnant women from the exposed group and half (52.2%) of the pregnant women from the unexposed group had low total gestational weight gain. The height of the pregnant women was < 1.45 m in 11.5 and 6.3% of the exposed and unexposed pregnant women, respectively. At the first visit (ANC1), almost all (99.6%) exposed pregnant women had body mass index (BMI) of < 18.5 kg/m^2,^ and 252 (98.8%) unexposed pregnant women had a BMI ≥ 18.5 kg/m^2^. Thirty-six (14.7%) and 21(8.2%) pregnant women had intestinal parasites among the exposed and unexposed pregnant women, respectively. All pregnant women in both groups were non-reactive for syphilis and hepatitis B virus but 11(4.5%) pregnant from the exposed group and 5(1.9%) pregnant women from the unexposed group were reactive for HIV test. Furthermore, 60 (24.5%) from the exposed group and 32 (12.5%) pregnant women from the unexposed group had anemia (Hemoglobin< 11 g/dl) during antenatal care visit one. Concerning the mode of delivery for the index pregnancy; half of the pregnant women in both groups gave delivery via spontaneous vaginal delivery, the rests were through cesarean section and instrumental. More than half (52.2%) of the newborns from the exposed group and two-third (64.7%) of the newborns among the unexposed group were females.

### Incidence and risk factors of low birth weight

In this study, the mean (± standard deviation) of birth weight was 2.96 (SD ± 0.45). The overall cumulative incidence of low birth weight was 14% (95%CI: 11.1, 17.4%). The cumulative incidence of low birth weight among the exposed pregnant women was 18.4% (95%CI: 14.1, 23.9%) and among the unexposed pregnant women, it was 9.8% (95%CI: 6.8, 14.2%). The difference in the incidence among these two groups was assessed using the chi-square (X^2^)-test and it was found that the difference in low birth weight incidence between the exposed and unexposed groups was statistically significant (*p*-value = 0.006).

Initially independent variables were assessed for their association with the dependent variable one by one. Those independent variables with *p*-value ≤0.2 were included in the log-bimodial model. Accordingly; maternal illiteracy, low monthly family income, lack of latrine utilization, and low diet diversity score were statistically significant risk factors of low birth weight. Illiterate pregnant women were 1.8 times more likely to give birth to newborns with low birth weight as compared to pregnant women with the educational status of above secondary school (ARR:1.8, 95%CI: 1.01, 3.3). Pregnant women who had monthly family income less than 50 US Dollar were 1.6 times higher to have newborns with low birth weight as compared to pregnant women with a monthly income of more than 167 US Dollar (ARR: 1.6, 95%CI: 1.07, 2.2). The utilization of latrine was a statistically significant factor for low birth weight. Pregnant women who utilized latrine were 53% less likely to have low birth weighted newborns (ARR: 0.47, 95%CI: 0.28, 0.78). Pregnant women who had a diet diversity score of less than five were 1.9 times more likely to have newborns with low birth weight as compared to pregnant women who had five and above diet diversity scores (ARR:1.9, 95%CI: 1.05,2.61) (Table 5). Multi-collinearity was checked using variance inflation factor (VIF) at < 8 but the VIF of all variables was less than two which means no multi-collinearity. Moreover, the interaction of the variables at a *p*-value of < 0.05 was assessed and there was no interaction. In the omnibus test, the likelihood ratio chi-square test indicated that the full model was a significant improvement in fit over a null (no factor) model (*p*-value< 0.001).

**Table 5:** Result of the log-binomial model to identify risk factors for low birth weight in Tigray regional state, Ethiopia, 2020

Variables		Low birth weight		CRR (95%CI)	ARR (95%CI)
		Yes (%)	No (%)		
Maternal height	≤1.45 m	3 (4.3)	41 (9.5)	0.15 (0.12, 1.8)	0.56 (0.19,1.64)
	>1.45 m (ref.)	67 (95.7)	389 (90.5)	1	1
Maternal educational status*	Illiterate	18 (25.7)	109 (25.3)	0.06 (0.57,1.99)	1.8 (1.01, 3.3)*
	Elementary school	16 (22.9)	109 (25.3)	0.96 (0.5,1.8)	1.89 (0.89,4.04)
	High school	20 (28.6)	108 (25.1)	1.17 (0.64,2.5)	1.86 (0.98,3.44)
	Above high school (ref.)	16 (22.9)	104 (24.2)	1	1
Maternal occupational status	Civil servant	25 (35.7)	84 (20)	2.63 (1.55,4.45)	2.69 (1.56,4.68)
	Farmer/daily worker	5 (7.1)	45 (10.7)	1.15 (0.46,2.88)	1.08 (0.43,2.69)
	Own business	18 (25.7)	60 (14.3)	2.64 (1.49,4.67)	1.75 (0.98,2.32)
	Housewife (ref.)	22 (31.4)	230 (54.9)	1	1
Monthly family income**	< 50 US Dollar	23 (32.9)	99 (23)	1.09 (0.68,1.76)	1.6 (1.07,2.2)**
	50–167 US Dollar	13 (18.6)	168 (39.1)	0.42 (0.23,0.76)	0.47 (0.28, 0.78)
	> 167 US Dollar (ref.)	34 (48.6)	163 (37.9)	1	1
Family size	≤4	50 (71.4)	267 (62.1)	1.44 (0.89,2.35)	1.44 (0.88,2.34)
	> 4 (ref.)	20 (28.6)	163 (37.9)	1	1
Utilization of latrine*	Yes	34 (48.6)	319 (75.6)	0.37 (0.24,0.57)	0.47 (0.28, 0.78)*
	No (ref.)	36 (51.4)	103 (24.4)	1	1
Fasting during pregnancy during a fasting period	Yes	27 (38.6)	195 (45.3)	0.79 (0.5,1.23)	1.12 (0.71,1.75)
	No (ref.)	43 (61.4)	235 (54.7)	1	1
Presence of diseases	Yes	2 (2.9%)	58 (13.5)	0.22 (0.05,0.86)	0.25 (0.06,0.98)
	No (ref.)	68 (97.1)	372 (86.5)	1	1
DDS*	Low (< 5)	35 (50)	226 (52.6)	0.92 (0.59,1.4)	1.9 (1.05,2.61)*
	Adequate (≥5) (ref.)	35 (50)	204 (47.4)	1	1
Alcohol drinking during pregnancy	Yes	6 (8.6)	84 (19.5)	0.43 (0.19,0.96)	0.42 (0.18,0.95)
	No (ref.)	64 (91.4)	346 (80.5)	1	1
Total gestational weight gain	Low	51 (72.9)	262 (60.9)	1.6 (0.98,2.6)	1.45 (0.9,2.32)
	Adequate (ref.)	19 (27.1)	168 (39.1)	1	1

* < 0.05, ** < 0.01, CRR: Crude Risk Ratio, ARR: Adjusted Risk Ratio, CI: Confidence Interval, ref.: reference category

## Discussion

The overall cumulative incidence of low birth weight was 14% (95%CI: 11.1, 17.4%). The incidence of low birth weight was 18.4% (95%CI: 14.1, 23.9%) among the exposed pregnant women and 9.8% (95%CI: 6.8, 14.2%) among the unexposed pregnant women. The difference in low birth weight incidence between the exposed and unexposed was statistically significant (*p*-value = 0.006). The incidence of low birth weight in this study was consistent with other studies conducted in Wolaita Sodo Hospital 15.8% [20], Felege Hiwot hospital 11.6% [21], Jimma Hospital 14.6% [22], and DebreTabor Hospital 12% [23].

On the other hand, the incidence of low birth weight in our study is lower than the incidence reported from studies conducted in DebreMarkos Hospital 21.6% [24], Northeast India 26% [25], and Nepal 23.6% [26]. The difference could be due to the lowest mean birth weight in Asia [26]. Again, the study period was in 2017, 2016 and 2012 for the study conducted in DebreMarkos Hospital, Northeast India and Nepal, respectively during which the burden of malnutrition was higher as compared to 2020. Incidence of this study is higher as compared to incidences reported from studies conducted in Butajira Hospital 8.9% [27], Ghana 9.7% [28], and Nigeria 7.3% [29]. The study in Butajira Hospital was cross sectional and secondary data were used from mothers’ medical card, in which birth weight of the newborns might not be recorded; hence, incidence of low birth weight could be underestimated. Similarly, the study conducted in Ghana used secondary data and the study conducted in Nigeria used DHS data which were collected from mothers’ medical cards or from the mothers’ recall. This can cause recall bias and births might not take place in health facilities which may have resulted in an underestimation of the incidence of low birth weight. But, our study was prospective cohort study, and institutional delivery was must which avoids recall bias and birth weight was measured and recoded using standard procedure. Overall, this study showed a high incidence of low birth weight which indicates poor progress to achieve the World Health Assembly (WHA) target of reducing the prevalence of low birth weight to 10.5% or below by 2025 [30]. It is strongly supported that a newborn’s weight at birth is an important marker of fetal health and nutrition. Newborns with low birth weight have a higher risk of dying in the first 28 days of life and even those who survive are more likely to suffer from stunted growth, lower intelligence quotient (IQ), and poor quality of life.

Furthermore, this study revealed that illiterate pregnant women were 1.8 times more likely to give birth to newborns with low birth weight as compared to pregnant women with the educational status of above secondary school. This finding was supported by studies conducted in South-East Ethiopia [31], North Wello zone [32], Tanzania [33], and Indonesia [34]. This could be due to the fact that maternal education promotes a positive attitude towards health-seeking behavior, acquisition of health and nutrition knowledge, and adherence to recommended feeding practices during pregnancy [35].

Our study further indicated that pregnant women who had monthly family income less than 50 US Dollar were 1.6 times higher to have newborns with low birth weight as compared to pregnant women with a monthly income of more than 167 US Dollars. This finding was consistent with studies conducted in Southeast Ethiopia [31], Southwest Ethiopia [36], NorthWest Ethiopia [21], and Bangladesh [37]. Pregnant women need two extra meals in addition to the basic three meals but pregnant women with low monthly income may not get the extra meals and their diet could be poor in terms of quantity and quality which may result in low birth weight.

In our study, lack of latrine utilization was also a statistically significant factor for low birth weight in which pregnant women who utilized latrine were 53% less likely to have low birth weighted newborns compared to their counterparts. Similar findings were reported from other studies conducted in Southwest Ethiopia [36], India [38]. Poor or no utilization of latrine could reflect fecal contamination of the local environment which in turn could result in a high incidence of infectious disease and intestinal parasites, thus high levels of nutrient mal-absorption in pregnant women and the possibility to have low birth weight newborns.

Finally, our study showed that pregnant women who had a diet diversity score of less than five were 1.9 times more likely to have newborns with low birth weight as compared to pregnant women who had five and above diet diversity scores. This finding was consistent with findings of other studies conducted in West Ethiopia [39], and Ghana ([40]. The diets of pregnant women in low and middle-income countries (LMICs) are monotonous and predominantly plant-based with little consumption of micronutrient dense animal source foods, fruits, and vegetables. Hence, such poor diet diversity is likely to be deficient in multiple micronutrients which in turn affects women’s health and nutrition which resulting a negative impact of birth weight [41].

The strength of our study includes prospective nature, control of confounding factors, and low loss to follow up. Nevertheless, there were limitations like micronutrients were not measured except hemoglobin for anemia, the possibility of recall bias during the assessment of the diet diversity score using the previous 24 h recall method. DDS was estimated for one day which might be better if it was done for two and above days. Caffeine from chocolate, and soft drink was not measured, and private health facilities were not included. Besides, it does not provide evidence for a causal relation.

## Conclusion

The incidence of low birth weight in the Tigray regional state was significantly higher among the under-nourished pregnant women. Again, maternal illiteracy, low monthly family income, lack of latrine utilization, and low diet diversity score were risk factors of low birth weight. It is then important to strengthen nutritional assessment and interventions during pregnancy periods with special attention for illiterate, and low monthly family income pregnant women. Again, there has to be a promotion of latrine utilization and consumption of diversified diets.

## Data Availability

All data generated or analyzed during this study are included in this published article.

## Abbreviations

ANC: Ante-Natal Care
CI: Confidence Interval
DDS: Dietary Diversity Scores
EDHS: Ethiopia Demographic Health Survey
LBW: Low Birth Weight
SPSS: Statistical Package for Social Science
VIF: Variance Inflation Factor
WHO: World Health Organization
